# Mycobactin analogue interacting with siderophore efflux-pump protein: insights from molecular dynamics simulations and whole-cell assays

**DOI:** 10.3389/frabi.2024.1362516

**Published:** 2024-05-08

**Authors:** Mousumi Shyam, Abhishek Thakur, Caroline Velez, Chris Daniel, Orlando Acevedo, Sanjib Bhakta, Venkatesan Jayaprakash

**Affiliations:** ^1^ Department of Pharmaceutical Sciences & Technology, Birla Institute of Technology, Ranchi, India; ^2^ Mycobacteria Research Laboratory, Department of Natural Sciences, Institute of Structural and Molecular Biology, Birkbeck, University of London, London, United Kingdom; ^3^ Department of Chemistry, University of Miami, Coral Gables, FL, United States

**Keywords:** mycobactin analogues, efflux-pump MmpL4, MmpL5, efflux-pump inhibitors, homology modelling, molecular docking, molecular dynamics

## Abstract

**Introduction:**

In response to continued public health emergency of antimicrobial resistance (AMR), a significant key strategy is the discovery of novel mycobacterial efflux-pump inhibitors (EPIs) as potential adjuvants in combination drug therapy. Interest in identifying new chemotypes which could potentially synergize with the existing antibiotics and can be deployed as part of a combination therapy. This strategy could delay the emergence of resistance to existing antibiotics and increase their efficacy against resistant strains of mycobacterial species. In recent decades, notable approaches have been accounted for EPI development and have resulted in the discovery of several EPIs including SQ109 and AU1235. In context, to accelerate newer EPIs with novel mode of action here we have discussed mycobactin analogues and highlighted *in silico* binding orientation with siderophore efflux-pump proteins MmpL4/5.

**Methods:**

3-(2-hydroxyphenyl)-5-(aryl)-pyrazoline series was investigated for whole-cell efflux-pump inhibitory activity against *Mycobacterium smegmatis* and *Mycobacterium abscessus.* Machine learning and molecular dynamics were performed to construct a MmpL4/5 complex embedded in a lipid bilayer to identify the putative binding site and to predict ligand-protein binding energetics. Furthermore, the identified HIT compound was investigated in synergistic assay with bedaquiline.

**Results:**

Compound **Il**, 2-(5-(4-fluorophenyl)-4,5-dihydro-1H-pyrazol-3-yl)phenol, was identified as the most potent efflux pump inhibitor against *M. smegmatis* in whole-cell efflux-pump investigation. Followed HIT **Il** employed against *M. abscessus* for efflux-pump inhibition investigations and notable whole-cell efflux-pump inhibitory profile has been observed. The theoretical investigations predicted compound **Il** to be selective towards MmpL4, with significant hydrogen bonding and π-π stacking interactions effectively blocking a critical Asp-Tyr dyad interaction network necessary for proton translocation. Compound **Il** with bedaquiline highlighted an additive profile against the *M. abscessus* pathogen.

**Conclusions:**

MD simulations and whole-cell assays are indicating potential development of compound **Il** as an adjunct to the existing therapeutic regimen against mycobacterial infections.

## Graphical Abstract

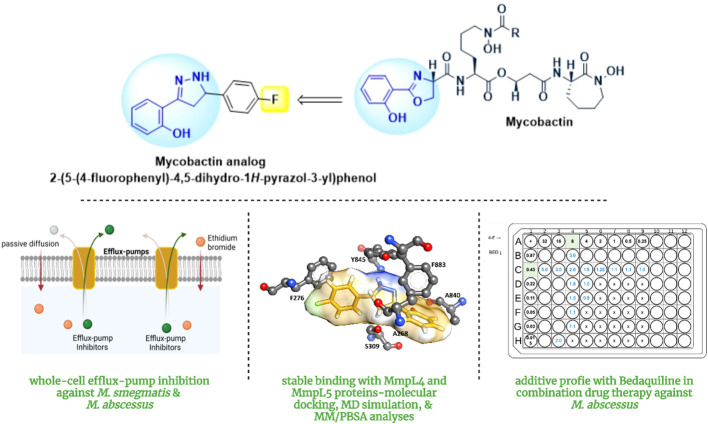

## Introduction

We are rapidly losing options to treat mycobacterial infections; aggressive resistance to “last resort” antibiotics poses a major threat to the drug-resistant (DR) strains of pathogens (Willyard, 2017). This rapidly emerging resistance has dire consequences with reports indicating that an estimated 700,000 deaths every year can be attributed to antimicrobial resistance (AMR), which is projected to reach a staggering 10.5 million per year by 2050 (O’Neill, 2014). A significant proportion of AMR mortality (27%) is attributable to *Mycobacterium tuberculosis* (Mtb)-caused by drug-resistant tuberculosis (DR-TB) (WHO, 2023) whereas *Mycobacterium abscessus*, a fast-growing non-tubercular mycobacterium (NTM), from the same *acenatobactor* family is becoming increasingly prevalent, particularly in individuals with congenital lung disorders such as cystic fibrosis (Johansen et al., 2020; Ganapathy and Dick, 2022).

**Figure 1:**
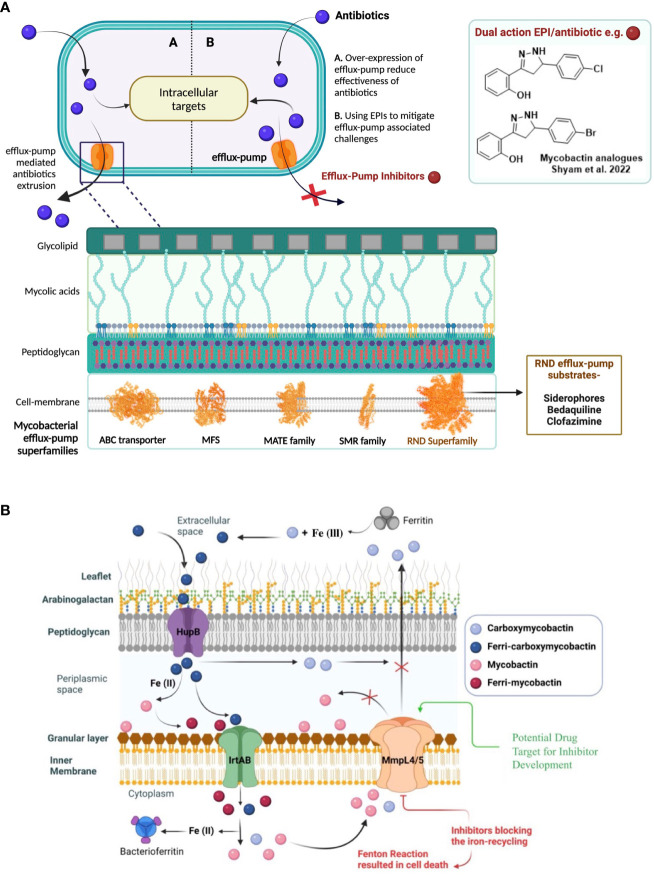
**(A)** Efflux-pumps mediated drug resistance in mycobacteria and as a possible solution, the discovery of potential efflux-pump inhibitors (EPIs) to tackle rapidly emerging antibiotic-resistance. 5- major efflux-pump families are available in mycobacteria i.e., ATP-binding cassette (ABC), major facilitator superfamily (MFS), multidrug and toxic compound extrusion family (MATE), small multidrug resistance family (SMR) and resistance-nodulation-division (RND). **(B)** Siderophores mediate iron-transportation is one of the key strategies of mycobacterial survival at host macrophages and virulence factor. To avoid excess siderophore-accumulation in bacterial compartment resulted into toxicity, mycobacteria recycle siderophores through MmpL4 and MmpL5 efflux-pump proteins to main iron-homeostasis.

Mycobacteria exploit different intrinsic mechanisms to resist drug therapy that include decreased membrane permeability of antibiotics, alteration or destruction of the antibiotic, mutation of the antibiotic target, and overexpression of efflux pumps (Tenover, 2006). Overexpression of efflux-pumps is one of this pathogen’s most crucial defense mechanism (Li et al., 2015). Mycobacteria contain the following five major “superfamilies” of efflux pumps: ATP-binding cassette (ABC), major facilitator superfamily (MFS), multidrug and toxic compound extrusion family (MATE), small multidrug resistance family (SMR) and resistance-nodulation-division (RND) (Webber and Piddock, 2003) (**Figure 1**). All these five classes of efflux pumps implicate resistance to different antibiotics in mycobacteria. The last decade has seen some substantial efforts in the evolving field of efflux-pump inhibitors (EPIs), leading to the identification of a few countable EPIs, including SQ109 and AU1235 (Pule et al., 2015).

In the context of novel EPI discovery, we would like highlight RND efflux-pump mediated siderophore recycling and iron-homeostasis mechanism in mycobacteria commonly shared by pathogenic-Mtb and opportunistic-NTMs (Shyam et al., 2021; Shyam et al., 2022a). Iron is an essential micronutrient that mycobacteria require for their colonization and growth; however, free iron is highly restricted in host environments (approximately 10^−24^ M in human serum and body fluids). During infection in the host macrophages, mycobacteria produce salicyl-capped siderophores to chelate and internalize iron from the host iron-binding proteins to support their biomachinery. In particular, a hydrophilic siderophore, termed carboxymycobactin (cMBT), is released into the extracellular environment to chelate Fe^3+^ to form the Fe-cMBT complex (**Figure 1**) (Shyam et al., 2021; Shyam et al., 2022a). The Fe-cMBT complex is transported into the periplasmic space to release free iron. The released iron in the periplasmic space gets bound to the cell wall-associated lipophilic siderophore mycobactin (MBT) forming Fe-MBT. The Fe-MBT complex is then transported into the cytoplasmic space and subsequently releases Fe^2+^ into the cytoplasm (Shyam et al., 2021; Shyam et al., 2022a). Finally, the siderophores get recycled into the periplasmic and extracellular space through the RND superfamily efflux transporter, mycobacterial membrane protein large 4 and 5 (MmpL4/5) (Shyam et al., 2021, Shyam et al., 2022a) To maintain the iron homeostasis in mycobacteria, extrusion of siderophore through MmpL4/5 is referred to as a critical mechanism, as earlier studies reported that disruption of siderophore recycling leads to the accumulation of active siderophore inside the mycobacterial cell, which results in self-poisoning via a Fenton reaction-mediated suicidal mechanism (Shyam et al., 2022a) (**Figure 1**). Therefore, disrupting siderophore recycling by inhibiting the efflux-pump protein MmpL4/5 is an excellent target mechanism to reverse the drug resistance in mycobacteria (Shyam et al., 2022a).

MmpL4/5 efflux pumps, other than siderophore transportation, are closely associated with bedaquiline (BDQ) resistance (anti-TB drug BDQ approved in 2012 for combination therapy against multidrug-resistant TB, MDR-TB) (Mahajan, 2013). Urgent action is therefore required if the problem of efflux-mediated resistance to anti-TB drugs is to be addressed. In continuation of our previous work (Shyam et al., 2022b), we have investigated thirteen other 3,5-diaryl-4,5-dihydro-1H-pyrazole members in this work (**Table 1**), in search of new EPI identifications. We have determined these analogues as remarkable mycobactin biosynthesis inhibitors against a wide range of mycobacterial species (Shyam et al., 2022b), and we hypothesize that these analogues might target MmpL4/5 efflux-pumps mediated siderophore recycling mechanism.

**Table 1:** ** **A conclusive summary of the relative fluorescence unit (RFU) noted from the EtBr accumulation assay against both *M. smegmatis* and *M. abscessus*.

Compound	R	RFU^#^ (Mean ± SD)	Organism
**Ia**	2-OH	56813.33 ± 3725.35^***^	*M. smegmatis*
**Ib**	3-OH	46255.33 ± 905.39^****^	
**Ic**	4-OH	47249 ± 2443.97^****^	
**Id**	2-CH_3_	72015 ± 5578.57^ns^	
**Ie**	4-CH_3_	52397 ± 7536.21^****^	
**If**	2-OCH_3_	51560.33 ± 2377.47^****^	
**Ig**	4-OCH_3_	58450.67 ± 452.37^**^	
**Ih**	3-OH, 4-OCH_3_	70642.33 ± 2215.24^ns^	
**Ii**	H	70271.67 ± 9116.65^ns^	
**Ij**	2-Cl	55702.67 ± 4145.32^***^	
**Ik**	2-F	57789 ± 2914.45^***^	
**Il**	**4-F**	**81378.67 ± 799.11** ^ns^	
**Im**	2-Br	50547.67 ± 2570.17^****^	
**Control**	Verapamil	75102.33 ± 3125.57	
**Il**	**4-F**	**26533.00 ± 423.08** ^****^	*M. abscessus*
**Control**	Verapamil	16658.66 ± 835.96	

#RFU calculated in EtBr whole-cell efflux pump assay,Statistical analysis: One-way anova followed by Tukey’s HSD using R-package, ** p < 0.01, *** p < 0.001, **** p < 0.0001, ns not significant.

Potent ones are highlighted by bold letters.

In the current study, we have integrated a high-throughput whole-cell efflux pump screening of mycobactin analogues against *M. smegmatis* and *M. abscessus* alongside a detailed computational investigation. This featured various methodologies, such as machine learning and molecular dynamics to elucidate the origin of the observed inhibition arising from the most potent candidate. This required the construction of a novel MmpL4/5 complex embedded in a lipid bilayer to identify the putative binding site and to predict binding energetics. In addition, a significant effort has been made to analyze drug-resistance reverse potentiality by evaluating the synergistic potentiality of the most potent candidate with TB-therapeutic, BDQ.

## Materials and methods

### Bacterial strains and culture media

The bacterial species used in this study are *M. smegmatis* mc^2^155 (ATCC 700084), and *M. abscessus* (NCTC 13031). Mycobacterial strains were cultured in Middlebrook 7H9 broth (MB7H9; BD Difco™) supplemented with albumin/dextrose/catalase (ADC, Remel™) enrichment or Glycerol-Alanine-Salt Tween80 (GAST) medium.

### Resazurin microtiter assay

REMA is a rapid colorimetric redox MIC indicator, when reduced to resorufin, the solution can be visibly seen as pink, indicating aerobically active bacterial cells. We performed REMA following our earlier protocol (Shyam et al., 2022b). A 0.01% (w/v) resazurin (Alfa Aesar) solution was used. *M. abscessus* was grown in a rotating incubator at 37°C in MB7H9 until OD_600_ ~1.0 then diluted to ~1x10^3^ cells/mL, per well. Chellex-100 defferrated GAST medium (iron-deficient media), was prepared and used on a triplicate set of plates as well as *M. abscessus* grown in MB7H9. The plates were grown in a static incubator (Genlab) at 37°C for 48 h, 30 μL of resazurin was added, then left in a static incubator at 37°C for 48 h before being read. All plates were covered and parafilmed.

### Whole-cell drug efflux pump accumulation assay

The assay was performed following our earlier reported methodology (Shyam et al., 2022b) by using early log phase cells of *M. smegmatis* and *M. abscessus* (OD_600_ ~ 0.4) and compounds were tested at sub-MIC concentration (0.25 x MIC) to ensure unaltered cell viability. Cells were collected by centrifugation and resuspended in an equivalent volume of 1 x PBS. The test samples comprised bacterial culture 10^7^ bacteria/mL in PBS along with 0.4% glucose as the source of energy for efflux pump activity. A stock of 0.5 mg/L ethidium bromide (EtBr) was used as the substrate for efflux pumps, and verapamil was used as the positive control at a concentration of 125 μg/mL for *M. smegmatis* and 256 μg/mL for *M. abscessus*. The experiment was performed in a 96-well plate that was placed in a fluorimeter (Biotek Synergy 2) and programmed with following parameters: excitation 540 nm, emission 620 nm, fluorescence gain 80, cycle of measurement every minute for a total period of 60 min at 37°C.

### Checkerboard assay

Individual MICs were determined by REMA and used for the checkerboard assay (Shyam et al., 2022b). A 2-fold dilution was conducted of BDQ and compound **Il** across a 96-well plate. *M. abscessus* was grown and resazurin added as per the REMA. The fractional inhibitory concentration index (FICI) value was calculated with the following formula:


$$\frac{A}{MIC_{A}}+\;\frac{B}{MIC_{B}}=FIC_{A}+\;FIC_{B}=FIC\;index$$

where, A and B are the MICs of each antibiotic in combination and MIC_A_ and MIC_B_ are the MICs of the antibiotic individually. A FICI < 0.75 is considered synergistic, between 0.75 and 4.0 is considered additive or indifferent, and a FICI > 4.0 is considered antagonistic.

### Protein preparation

Starting Cartesian coordinates for the MmpL4 and MmpL5 receptors were constructed using both homology modelling (Waterhouse et al., 2018) and AlphaFold (Jumper et al., 2021). In this study, the homology model constructed for the MmpL4 system was created using SWISS-MODEL (Waterhouse et al., 2018). A 3D model of the given target protein sequence (MmpL4) is based on its alignment to a protein of known structure (template). In the current work, the MmpL4 3D structure was built upon a sub-family protein MmpL3 crystal structure (PDB ID: 6AJG (Zhang et al., 2019) of resolution 2.6 Å with an identity of 24.5%. In contrast to homology modelling, Google’s deep learning-based AlphaFold (Jumper et al., 2021) was used to derive the 3D structure of an entire sequence of MmpL4 and MmpL5 for the *M. tuberculosis* strain (ATCC 25618/H37Rv) (Uniprot Id: P9WJV3 and P9WJV1).

### Molecular docking

AutoDock 4.2 program (Morris et al., 2009) was used in this study to predict ligand binding modes by using a combination of the Lamarckian Genetic Algorithm and empirical force field parameters. The MmpL4/5 protein structure (apo state) predicted by AlphaFold was subjected to minimization and equilibration as described in the molecular dynamics section. The equilibrated MmpL4/5 receptor then provided the starting Cartesian coordinates for the calculations. A grid box was generated at the center of the protein and was large enough to encompass the entire folded structure [region where the per-residue confidence score predicted by AlphaFold was very high] with a size of 90 grid points at each axis (x, y, and z). All AutoDock default parameters were utilized, with the exception that the exhaustiveness of the global search was set to 100.

### Molecular dynamics

Molecular dynamics (MD) simulations were performed on the MmpL4 and MmpL5 protein-ligand complexes. The PACKMOL-Memgen software (Schott-Verdugo and Gohlke, 2019) distributed with AmberTools23 was used to embed the enzyme complexes into a lipid bilayer consisting of 1-stearoyl-2-palmitoyl-sn-glycero-3-phopshoethanolamine (SPPE), 1,3-bis(1-oleoyl-2-palmitoyl-sn-glycero-3-phospho)-sn-glycerol (OPOPCL), 1-palmitoyl-2-oleoyl-sn-glycero-3-phosphoinositol (POPI), and 1-palmitoyl-2-myristoyl-sn-glycero-3-phosphoinositol (PMPI) lipids, in a 27:38:2:33 ratio (**Supplementary Figure S2**), respectively (Li et al., 2023; Vasyankin et al., 2024). These enzyme-ligand-lipid-bilayer systems, referred to as a complex, were solvated explicitly using a TIP3P cuboid box that enclosed the system based on its van der Waals boundary, and the overall charge of the complex was neutralized by adding a suitable number of potassium cations. Each system was subsequently solvated in a 0.15 M solution of KCl. The generalized Amber force field (GAFF2) (Jorgensen et al., 1983; Wang et al., 2004; Vassetti et al., 2019; He et al., 2020) was used to parameterize the inhibitor, the protein topology file was created using the ff14SB force field (Maier et al., 2015), and the lipid-bilayer was generated with Lipid21 parameters (Dickson et al., 2022). All systems were subjected to a five-step minimization protocol performed using the sander Amber22 engine. The water molecules and ions were initially minimized while the complex residues were harmonically restrained with 25 kcal mol^-1^Å^-2^ using the steepest descent (SD) algorithm for 5,000 steps, followed by 5,000 steps of conjugate gradient (CG) minimization. Next, water molecules and ions were minimized while the complex residues were harmonically restrained with 5 kcal mol^-1^Å^-2^ using the using the SD algorithm for 5,000 steps, followed by 5,000 steps of CG minimization. Subsequently, solvent molecules and lipid bilayer residues were minimized while the enzyme-ligand residues were harmonically restrained with 5 kcal mol^-1^Å^-2^ using the SD algorithm for 5,000 steps, followed by 5,000 steps of CG minimization. This was followed by harmonically restraining only the enzyme with 5 kcal mol^-1^Å^-2^ and allowing the rest of the system to undergo 5,000 steps of SD minimization, followed by 5,000 steps of CG minimization. Lastly, the entire system was minimized without restraints using the SD algorithm for 5,000 steps, followed by 5,000 steps of CG minimization. Thereafter, the system underwent a three-step equilibration protocol using mixed CPU and GPU-enabled Amber22 engines. To prime the system velocities, the system was gradually heated from 0 to 100 K, with harmonic restraints of 5 kcal mol^-1^Å^-2^ on the enzyme-ligand residues, in four 25 K increments using a constant NVT ensemble over 5 ps each with a Langevin thermostat, a temperature coupling value of 1.0 ps, and a collision frequency (γ) of 10.0 ps^-1^, 7.5 ps^-1^, 5.0 ps^-1^, and 1.0 ps^-1^, respectively. An additional heating step from 100 K to 310 K, with harmonic restraints of 5 kcal mol^-1^Å^-2^ on the enzyme-ligand residues, using a constant NPT ensemble over 115 ps with a Langevin thermostat, a temperature coupling of 1.0 ps, and a γ of 1.0 ps^-1^ was performed. Finally, to equilibrate periodic boundary conditions (PBC) 5 ns stepwise simulations were performed for over 50 ns using a constant NPT ensemble at 310 K and 1 atm with the temperature and pressure coupling values set to 1.0 ps with identical Langevin parameters as the second equilibration phase. The last 5 ns of the equilibration phase were used to compute boost potentials for the subsequent 100 ns accelerated molecular dynamics (aMD) simulations using a constant NVT ensemble at 310 K (Gangadevi et al., 2021; Jumper et al., 2021; Gangireddy et al., 2022). Degrees of freedom boosted per system and their respective values are provided in **Supplementary Table S1**. Long-range electrostatics were accounted for by using the particle mesh Ewald, all covalent bonds involving hydrogen atoms were constrained with the SHAKE algorithm, periodic boundary conditions were enforced using a nonbonded cutoff distance of 12 Å, and a time step of 1.0 fs was utilized. All computational analysis was performed with the cpptraj and ptraj programs available in the AmberTools23 suite and custom python scripts (Roe and Cheatham, 2013). The root-mean-square deviations (rmsd’s) and fluctuations (rmsf’s) were calculated to monitor the structural stability of each simulation, and are provided in the **Supplementary Figure S3**.

## Results

### Investigation of *Mycobacterium smegmatis* efflux-pump inhibition by mycobactin analogues

**Figure 2:**
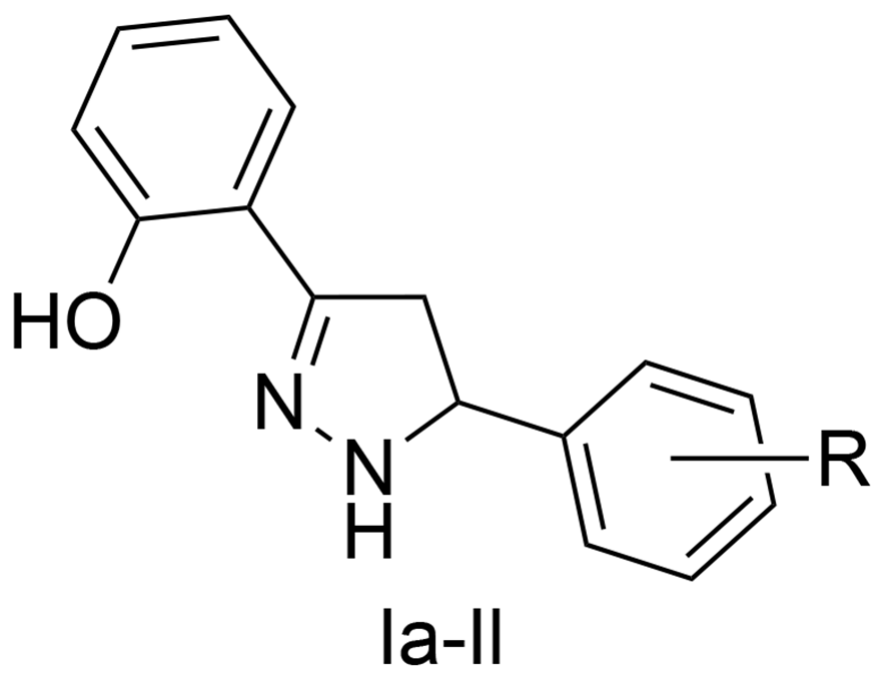
Basic chemotype of the pyrazoline analogues.

These analogues are termed mycobactin analogues as they partially mimic the chemical framework of the iron-scavenging siderophore mycobactin and are identified as potential inhibitors of mycobactin biosynthesis (Shyam et al., 2022b). In our recently published research article, the steps of chemical synthesis and structural analysis (including ^1^H NMR, ^13^C NMR, ESI-MS, and HR-MS), and antimycobacterial profiles against *M. tuberculosis* H37Rv, *M. smegmatis*, *Mycobacterium bovis* BCG and *Mycobacterium aurum* are reported for these analogues (Shyam et al., 2022b). We have used *M. smegmatis* to examine the suppression of the whole-cell efflux pump by mycobactin analogues against mycobacteria. A non-virulent mycobacterial strain, *M. smegmatis* mc^2^155, is frequently employed as a surrogate model for the screening of recently identified efflux pump inhibitors to avoid the complexity associated with *M. tuberculosis* in the early drug discovery stage (Johnson et al., 2020). However, pathogenic, non-pathogenic, and NTM categories all-share siderophore biosynthesis and recycling pathways (Shyam et al., 2021; Shyam et al., 2022a). In the fluorometric EtBr experiment, EtBr serves as the efflux substrate and fluoresces when in combination with DNA (Lepecq and Paoletti, 1967) (EtBr is approximately 30-fold more luminous intracellularly than in the extracellular environment). Therefore, as EtBr builds up inside the cell, fluorescence will increase over time if the substance being tested is an efflux inhibitor. In a discussion of comparative efficacy, the prevalent efflux-pump inhibitor (EPI) verapamil (VP) is employed as the positive control. Minimum inhibitory concentration (MIC) values were calculated against *M. smegmatis* as reported in our earlier study (Shyam et al., 2022b). We investigated efflux-pump inhibition by determining the relative fluorescence unit (RFU) at the sub-MIC dose concentration over a 60 min time-window for individual candidates (**Table 1**, **Figure 2**, **Supplementary Figure S4**).

In the EtBr whole-cell efflux pump assay, it has been noted that the substitution of fluorine at the *para* position of the benzene ring on the 3-(2-hydroxyphenyl)-5-(aryl)-pyrazoline scaffold (compound **Il**), yielded high efflux pump inhibitory activity in comparison to verapamil (a known efflux pump inhibitor) (**Table 1**). During lead optimization of the inhibitor, bioisosteric replacement of fluorine with oxygenated functional groups (hydroxy and alkoxy) is a common path adopted in structure-based drug design. As oxygen and fluorine have similar sizes and share many properties, the addition of a hydroxy or alkoxy group means incorporating a hydrogen bond donor group and increasing the polarizability (as fluorine is highly electronegative). However, such bioisosteric replacement of fluorine at the *para* position of a benzene ring with a hydroxy or methoxy group (compounds **Ic** and **Id**, respectively) did not increase the potency of this pyrazoline scaffold as efflux pump inhibitors. As we know, fluorine is more electronegative than oxygen, so it has a stronger electron-withdrawing effect and increases the lipophilicity of the compound. This clarifies that the fluorine atom replacement is the most plausible reason for the increased inhibitory activity. In addition, the fluorine atom replacement prohibits the scaffold from developing a metabolic oxidation reaction that is advantageous over other bioisosteric substitutions.

### Investigation of antimycobacterial activity and efflux-pump inhibition against *Mycobacterium abscessus* by the identified HIT, compound–Il

The intrinsic and acquired resistance of *M. abscessus* to commonly used antibiotics limits the therapeutic options to treat infection caused by this pathogen (Johansen et al., 2020). Moreover, a lackluster effort has been noted for rationale design inhibitors development against NTMs. We investigated HIT candidate **Il** against *M. abscessus* in our search for novel antibiotics with novel mechanisms of action i.e., by disrupting iron-homeostasis (Bythrow et al., 2022; Mudde et al., 2022) against NTM. Both MB7H9 broth, a traditional mycobacteria growth medium that is iron-enriching, and GAST, an iron-deficient environment, were used to assess compound **Il**’s MIC against *M. abscessus* in a comparative manner to understand putative target preferentiality (Shyam et al., 2022b). The MICs in MB7H9 and GAST medium were noted 256 g/mL and 8 g/mL, respectively, showing a 32-fold higher inhibition in an iron-deficient condition over an iron-supplemented medium and emphasizing antimycobacterial efficacy associated with the suppression of mycobactin synthesis. The primary hypothesis behind investigating the designed mycobactin analogues in iron-rich and iron-deprived medium to understand the target preferentiality of these analogues. We were more interested to identify a HIT candidate which could potentially inhibit the growth of mycobacteria in iron-deprived medium and showing less/no lethal effect in iron-rich medium as siderophore biosynthesis occurs at iron-deprived condition.

**Figure 3:**
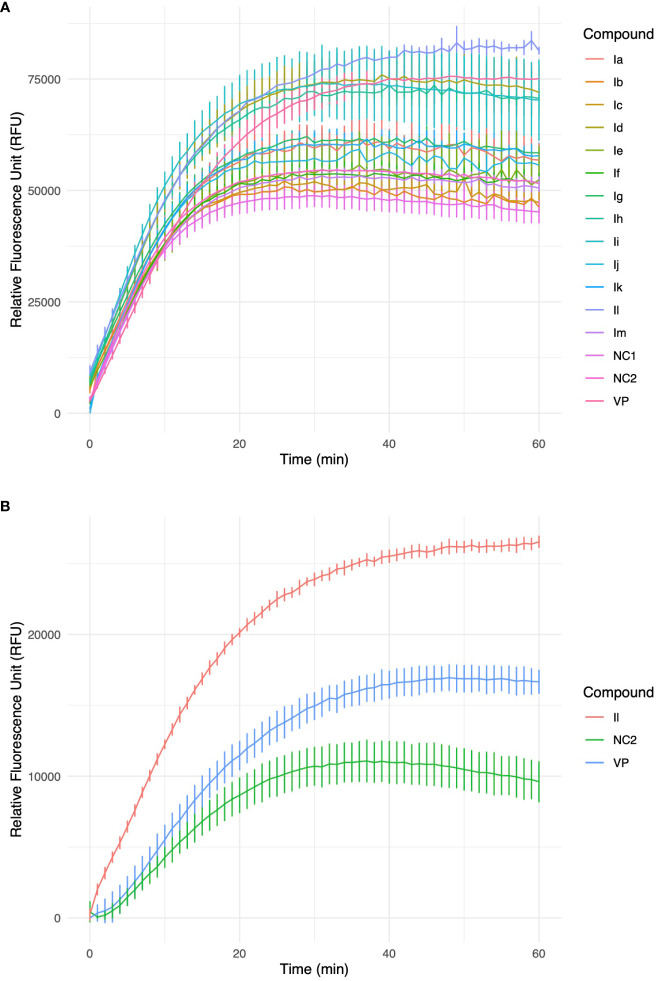
**(A)** Accumulation of ethidium bromide (EtBr) within *M. smegmatis* cells over a 60-minute timeframe in the presence of 3-(2-hydroxyphenyl)-5-(aryl)-pyrazolines analogues, VP-verapamil, NC-negative control (no inhibitor). Experiment was performed in biological triplicate (n=3), values represent the mean ± SD. **(B)** Furthermore, identified HIT, **Il** was investigated in an accumulation of EtBr assay within *M. abscessus* cells over a 60 min timeframe. Experiment was performed in biological triplicate (n=3), values represent the mean ± SD.

Excellent efflux-pump inhibition was observed following examination of whole-cell efflux-pump inhibition in *M. abscessus* at a sub-MIC (**Table 1** and **Figure 3**). Findings indicate compound **Il** showed greater efficacy against *M. abscessus* relative to VP over *M. smegmatis*. Interestingly, both verapamil and compound **Il** produced lower RFUs in *M. abscessus* than *M. smegmatis*, and we hypothesized that this occurrence could potentially be due to the relative concentration of positive controls and test compounds in relation to the increased number of MmpL proteins found in *M. abscessus* in comparison to *M. smegmatis* (31 and 17 *mmpL* genes, respectively (Deshayes et al., 2010).

### Computational prediction and validation of the putative drug target for the HIT identified from the whole-cell efflux pump

As MmpL4 and MmpL5 oversee siderophore recycling, we hypothesized that a pocket for mycobactin binding should exist in the MmpL4/MmpL5 complex present in mycobacteria that possesses the spatial geometry required to accommodate the signature hydroxyphenyloxazoline chemical block of mycobactin. Utilizing this insight, we designed compounds with a similar hydroxyphenyloxazoline layout, hypothesizing that they could potentially bind to and interfere with the MmpL4/5 efflux-pump proteins. Through cell-based experiments, we identified compound **Il** as a promising inhibitor of *M. abscessus*. However, while these assays provided valuable initial insights, they did not offer a detailed explanation for the superior performance of compound **Il**. Consequently, an in-depth computational investigation was performed to elucidate compound’s **Il** inhibitory profile. To date, no crystal structures have been reported for the MmpL4/5 proteins. As a result, the initial structural coordinates of the MmpL4/5 complex were derived using the neural network-based model AlphaFold. Developed by Google DeepMind, AlphaFold has been shown to predict protein structures from amino acid sequences with extraordinary atomistic accuracy, even in the absence of a homologous structure (Jumper et al., 2021).

To validate the AlphaFold-predicted structure, an additional homology model of MmpL4 was constructed using a reported experimental structure of MmpL3 (Zhang et al., 2019) and the SWISS-MODEL software (Waterhouse et al., 2018). Visual comparison of the two generated structures suggested that multiple regions within the SWISS-MODEL generated homology model did not properly fold (**Supplementary Figure S1**). However, both methods did generate a similar active site region where the AlphaFold per-residue confidence score was greater than 90. Nearby regions also appeared to be properly folded with AlphaFold giving per-residue confidence scores ranging from 70 to 90 for both the predicted MmpL4 and MmpL5 structures. As such, the AlphaFold generated MmpL4/5 models were adopted in this study to identify the inhibitor binding site and predict the ligand binding pose. It should be noted that two AlphaFold predicted regions were not properly folded, residues 1-18,477-710 and 931-967, but were located far away from the putative ligand binding site; consequently, these residues were truncated and capped during the subsequent MD simulations (**Supplementary Figure S1**).

**Figure 4:**
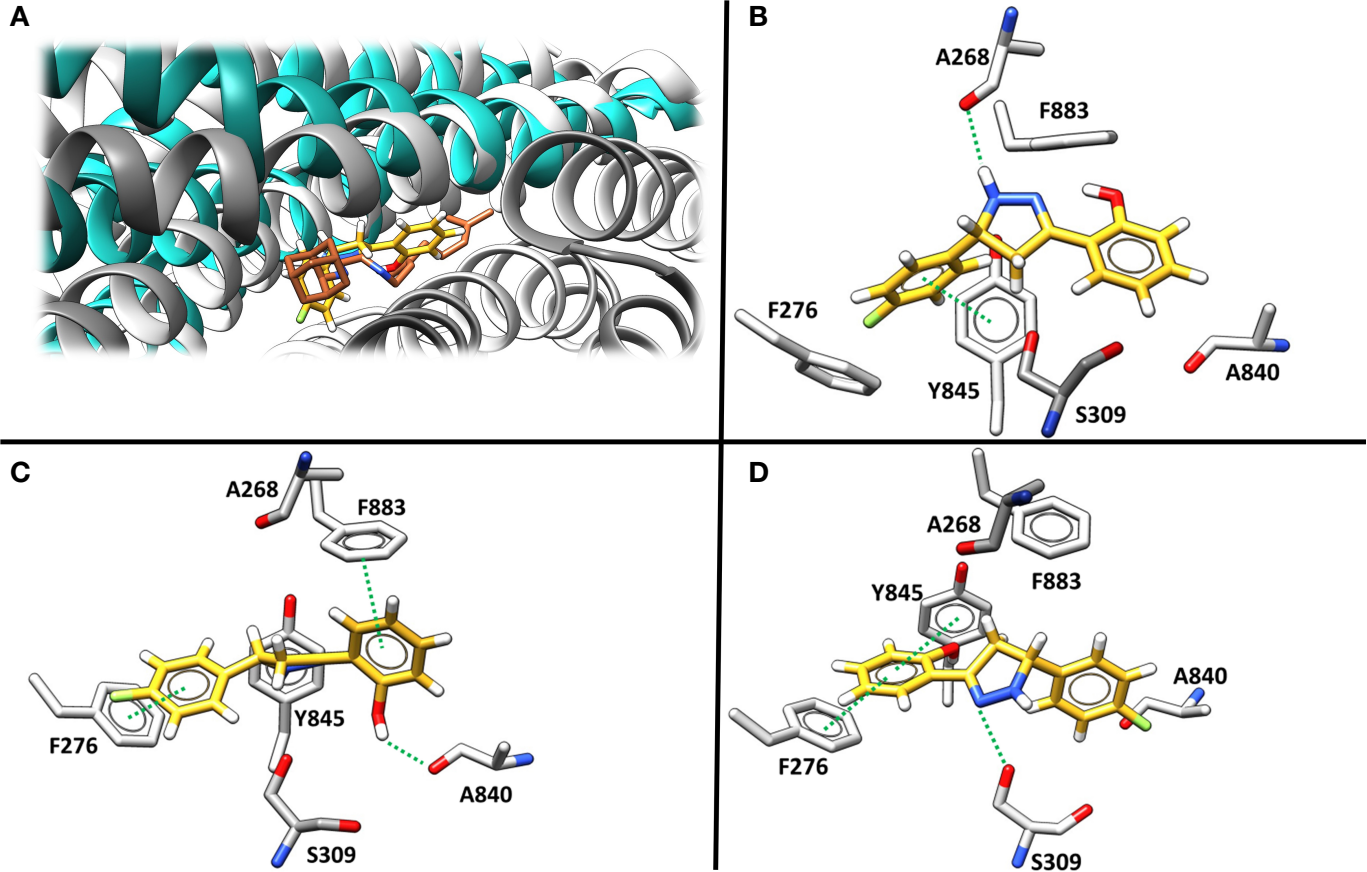
**(A)** The superimposed binding mode of inhibitor SQ109 (brown) co-crystallized against MmpL3 (PDB ID: 6AJG (Zhang et al., 2019) (blue) against the docked pose for compound **Il** (yellow) bound at the active site of MmpL4 system (grey). **(B–D)**. illustrate three unique predicted docking conformations (orientation 1, 2 and 3, respectively) for compound **Il** bound at the MmpL4 protein active site.

**Figure 5:**
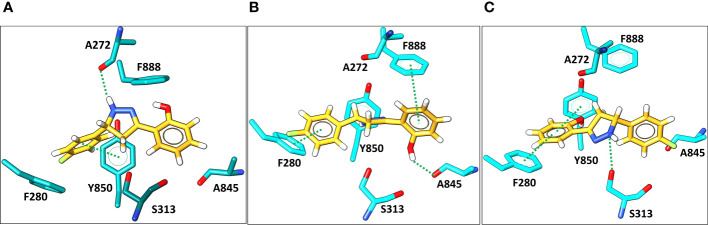
Docking predictions of three different binding conformations (orientation 1, 2, and 3, in **A–C**, respectively) of compound **Il** (yellow) bound within the MmpL5 (cyan) protein active site.

Molecular docking was then applied to predict the location of the ligand binding pocket and the potential ligand poses using the unbound (i.e., apo) AlphaFold-predicted structures of the transporters. In this study, blind docking was carried out using compound **Il** and the putative binding pocket identified in the MmpL4 protein was homologous to the MmpL3 active site (**Figure 4A**). The agreement between the binding site of the co-crystallized structure of the MmpL3 inhibitor (SQ109) and docked pose of compound **Il** provided high confidence in the docking protocol. The docking study identified three different binding poses within the same binding pocket with each pose possessing different electrostatic interactions with the active site residues (**Figures 4B–D**). Given the positive agreement between the experimental MmpL3 and the predicted MmpL4 structure of the ligand binding site, one may assume that family-member MmpL5 would possess a similar binding site. To verify, compound **Il** was investigated in MmpL5 using an identical docking procedure. The high structural similarity between the MmpL isoforms resulted in the same active site residues interacting with the ligand in a nearly identical fashion (**Figure 5**).

Given the known limitations of docking scores for estimating binding affinities and the lack of an encompassing microenvironment, a more computationally demanding *in silico* approach was required to compute more precise binding affinities. Accordingly, the most favorable binding orientations (i.e., 1, 2, and 3 given in **Figures 4**, **5**) for compound **Il** were examined in a complex of MmpL4 and MmpL5 transporters embedded within a lipid bilayer composed of 1-stearoyl-2-palmitoyl-sn-glycero-3-phopshoethanolamine (SPPE), 1,3-bis(1-oleoyl-2-palmitoyl-sn-glycero-3-phospho)-sn-glycerol (OPOPCL), 1-palmitoyl-2-oleoyl-sn-glycero-3-phosphoinositol (POPI), and 1-palmitoyl-2-myristoyl-sn-glycero-3-phosphoinositol (PMPI) lipids, in a 27:38:2:33 ratio respectively (discussed in detail in the method section). The model strived to mimic the transporters’ native lipophilic environment within the inner membrane (**Supplementary Figure S2**). Instead of relying on rigid-body docking methods, the embedded complexes were simulated using biased accelerated molecular dynamics (aMD) (Hamelberg et al., 2004; Gedeon et al., 2015) to account for any structural changes of the transporters that could occur as a result of both the protein-ligand binding event and the encapsulating lipid bilayer. The structural stabilities of the MmpL4/5-**Il** complexes were monitored by computing the root-mean-square deviation (RMSD) and root-mean-square fluctuation (RMSF) using the backbone protein atoms from each aMD trajectory (**Supplementary Figure S3**). A steady RMSD plot was observed after ~10 ns of aMD simulations of the 100 ns trajectories for all complexes, suggesting stable complexes (**Supplementary Figure S3A**).

To better estimate binding affinities for compound **Il**, the resulting trajectories were leveraged to compute binding affinities using the MM/PBSA method (Miller et al., 2012; Greene et al., 2016; Wang et al., 2016; Xiao et al., 2017). Comparison of the computed binding free energies, ΔG*_bind_*, for all three orientations of compound **Il** bound to the MmpL4 and MmpL5 complexes found that orientation-3 bound to MmpL4 possessed the most favorable ΔG*_bind_* of -12.5 kcal/mol (**Table 2**). Interestingly, decomposing the MM/PBSA energetic contributions into van der Waals and electrostatics interactions and polar and nonpolar contributions to the solvation free energies (E*_vdw_*, E*_el_*, G*_pol_* and G*_np_*, respectively) found the largest differences in the total ΔG*_bind_* came from the E*_el_* and G*_pol_* contributions as the E*_vdw_* and G*_np_* gave comparable energetic values for all three binding orientations within both receptors.

**Table 2:** The binding free energy in kcal/mol obtained via MM/PBSA method for three possible orientations of compound II bound to MmpL4 and MmpL5, as shown in **Figures 4B–D** and **5A–C**.

Protein	Orientation	E*vdW*	E*el*	G*pol*	G*np*	ΔG*bind*
	1	-39.7 ± 2.3	-20.4 ± 3.9	71.6 ± 42.6	-3.3 ± 0.08	8.2 ± 43.0
MmpL4	2	-34.2 ± 3.2	-14.6 ± 5.3	67.3 ± 30.0	-3.4 ± 0.12	15.2 ± 29.2
	3	-38.8 ± 3.1	-21.6 ± 8.6	51.3 ± 9.9	-3.4 ± 0.09	-12.5 ± 11.6
	1	-39.4 ± 2.9	-14.1 ± 2.4	85.0 ± 33.8	-3.2 ± 0.10	28.4 ± 32.6
MmpL5	2	-38.8 ± 2.8	-17.6 ± 7.3	77.7 ± 34.4	-3.4 ± 0.08	17.9 ± 34.2
	3	-38.5 ± 3.8	-8.9 ± 4.1	73.1 ± 32.7	-3.3 ± 0.14	22.3 ± 34.0

**Figure 6:**
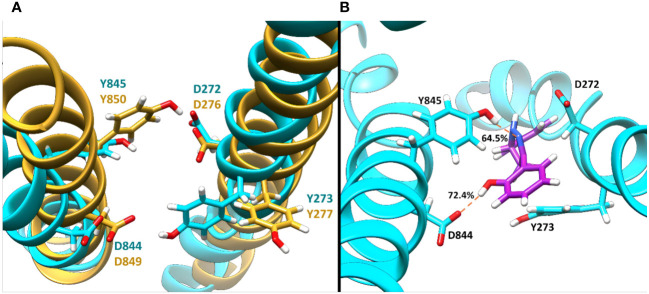
The representative binding site orientation and percent hydrogen bonding computed over the entire 100 ns aMD trajectory of the lowest energy structure of Compound **Il** in orientation-3 bound to MmpL4 and MmpL5. **(A)** Superposition of the lowest energy structure of bound MmpL4, shown in *cyan*, and MmpL5, shown in *gold*, sans ligand in orientation-3. **(B)** Lowest energy structure of the binding site of MmpL4, shown in *cyan*, complexed with compound **Il** in orientation-3, shown in *purple*.

To get further structural insight from the MD simulations, the lowest energy binding poses for the MmpL4/5-**Il** complexes were examined, and it was found that the ligand adopted a different conformation when compared to the initial binding pose predicted by molecular docking methods (**Figures 4B–D**, **Figure 6**). Of particular importance, as previously indicated in the literature, were two particular Asp-Tyr residue pairs, i.e., Asp272-Tyr845 and Asp844-Tyr273 in MmpL4, as these dyads are located in the active site and conserved across the transmembrane domains of the MmpL transporter family, e.g., Asp276-Tyr850 and Asp849-Tyr277 in MmpL5, with the exception of MmpL7 (Bernut et al., 2016; Zhang et al., 2019)

Studies employing mutational analysis have linked mutation of Asp-Tyr dyads in MmpL3 to the inhibition of mycobacteria growth, postulating that these Asp-Tyr residue pairs play a critical role in facilitating proton translocation across the MmpL family (Bernut et al., 2016). Within MmpL3, two sets of Asp-Tyr dyads are located at the center of the transmembrane domain, specifically in the middle region of α-helices 4 and 10, prompting structural stabilization of the helices via formation of hydrogen bonds (Bolla, 2020). Interestingly, the location of the Asp-Tyr dyads in MmpL3 is analogous to that of the Asp-Asp-Lys triad in AcrB, which is known to be involved in the proton relay network, and with whom MmpLs share homologous sequences. AcrB, an acridine resistance complex is a resistance-nodulation-cell division transporter multidrug efflux pump that is involved in the virulence and pathogenesis of various bacterial pathogens (Du et al., 2018; Fay et al., 2019) Similar to MmpLs, AcrB is hypothesized to be localized to the inner membrane (Fanelli et al., 2023). Considering the homology between MmpLs and AcrB, and the high sequence and structural similarity amongst MmpL isoforms, the dynamics of the binding active site pocket of MmpL4/5 are expected to be similar to that of MmpL3. These interactions are crucial for understanding the binding mechanism and potential inhibitory effects of compound **Il** within MmpL4/5. As a result, probing molecular interactions native to the protein-ligand binding event may aid in rationalizing the preference of compound **Il** for MmpL4.

The disruption of the Asp-Tyr dyads interaction network not only detrimentally affect the structure and stability of the protein, but also compromise the function of the transporter. Consequently, ligand orientations that fail to maximize interactions amongst Asp-Tyr dyads are energetically destabilizing and predicted to be poor binders. Energetically, compound **Il** positioned in orientation-3 within MmpL4 yielded the most favorable binding affinity, suggesting this specific orientation facilitates the ligand in maintaining stabilizing interactions with adjacent active site residues (**Table 2**). To identify the favorable electrostatic interactions that compound **Il** maintained within the active site of MmpL4, hydrogen bonding occupancies were monitored for the full trajectories, with special consideration placed on ligand interactions with Asp-Tyr dyads (**Table 3**). Detailed examination of the binding site within MmpL4 revealed compound **Il**, when in orientation-3, is positioned between the Asp-Tyr dyads, i.e., D272-Y845 and D844-Y273, which allows the compound to interact with the residues of both Asp-Tyr dyads (**Figure 6B**). As a result, the positioning of compound **Il** allows it to act as a pseudo replacement for an Asp-Tyr dyad interaction, which contributes to the more energetically favorable electrostatic contributions that are reflected in the E*_el_* and G*_pol_* terms from the MM/PBSA calculations. Unlike orientations 1 and 2, orientation-3 positioned the pyrazoline ring of compound **Il** near Y845(64.5%) while its hydroxyphenyl moiety was in close contact with D844 (72.4%) (**Figure 6B**), maximizing hydrogen bonding interactions within the Asp-Tyr network (**Table 3**). To a lesser extent, compound **Il** in orientation-3 maintained hydrogen bonding interactions with D272 (15%), with no hydrogen bonding interactions determined between the ligand and Y273, which is the complementary tyrosine residue of the D844-Y273 dyad (**Table 3**).

**Figure 7:**
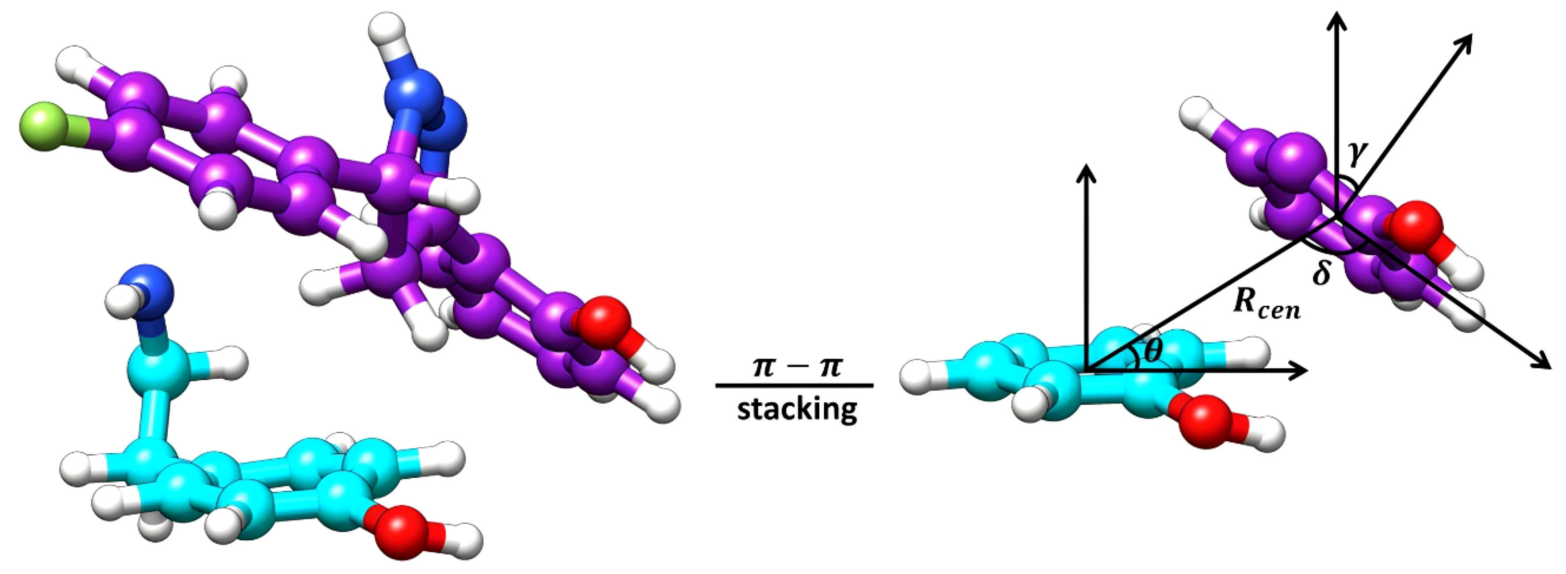
Graphical representation of the essential features for the computation of π-π stacking interactions. The lowest energy structure of Compound **Il**, colored *purple*, in orientation-3 and a tyrosine residue (Y273), colored *cyan*, of MmpL4 represent the surface planes being evaluated. The *R_cen_* is the distance between the planes, *θ* is the angle between the center of mass (COM) vectors and the tyrosine surface plane, δ is the angle between the COM vectors and the ligand surface plane, and *γ* is the dihedral angle between the planes.

Considering the positioning of compound **Il’s** hydroxyphenyl ring is vastly different in orientation-3 than alternative orientations, additional analysis was performed to elucidate if dispersion interactions, such as π-π stacking, was a contributing factor to the energetic favorability of compound **Il** when in orientation-3. Within proteins, π-π interactions generally have intermolecular distances ranging from ≤ 5.6 Å and dihedral angles, *γ*, ranging from 50° to 90° for non-substituted aromatic rings such as phenyls while substituted aromatic rings, like hydroxyphenyls slightly shift these ranges (Zhao et al., 2015). To account for π-π stacking interactions, normalized vectors centered on the mass of the hydroxyphenyl rings of the ligand and Y273 were used to compute the angle between them while centers of mass were leveraged to compute intermolecular distances (**Figure 7**) (Zhao et al., 2015; Hayatshahi et al., 2018; Somai et al., 2023). To estimate for π-π stacking interactions, a distance cutoff of ≤ 5.0 Å denoted by *R_cen_* and an angle cut-off of 0° to 180° was utilized, resulting in compound **Il** when in orientation-3 exhibiting stacking behavior for 81.8% of the trajectory while orientations 1 and 2 yielded dismal results of 9.5% and 0.0%, respectively. Notably, analysis of the lowest energy structure of the MmpL4-**Il** complex in orientation-3 revealed the hydroxyphenyls of the compound and Y273 maintained an intermolecular distance of 4.5 Å and a dihedral angle of 45.6°, supporting the notion of π-π stacking interactions, particularly T-shaped, being prominent within the protein-ligand binding event. Given the stacking behavior of compound **Il** in orientation-3 is almost an order of magnitude greater than alternative positions, the stacking analysis suggests only orientation-3 allows the compound to maintain π-π stacking interactions that further aid in promoting the binding event. Unsurprisingly, investigation into alternative positions revealed orientations 1 and 2 in MmpL4/5 failed to maintain notable hydrogen bonds with both Asp-Tyr dyads, mostly engaging with a sole aspartate residue from an Asp-Tyr dyad, i.e., D272-Y845 in MmpL4 and D276-Y850 in MmpL5 (**Table 3**). Thus, only compound **Il** when in orientation-3 maximized all Asp-Tyr dyad interactions through a combination of electrostatic and van der Waals forces, suggesting the ligand’s chemical framework is capable of effectively targeting MmpL4 over MmpL5.

**Table 3:** Computed percent hydrogen bonding between Compound II and Asp-Tyr dyad binding site residue pairs in MmpL4 and MmpL5 over the 100 ns MD trajectory.

Protein	Orientation	Asp-Tyr Dyad 1		Asp-Tyr Dyad 2	
		*D272*	*Y845*	*D844*	*Y273*
	1	81.4	52.2	–	–
MmpL4	2	5.5	–	–	–
	3	15.0	64.5	72.4	–
		*D276*	*Y850*	*D849*	*Y277*
	1	83.7	–	–	–
MmpL5	2	27.3	0.28	5.4	13.6
	3	–	–	–	–

### Compound Il in combination drug therapy with BDQ

It has already been highlighted that the newly discovered anti-TB drug BDQ for the therapeutic intervention of multi- and extreme-drug-resistant TB patients confers efflux-mediated resistance, indicating the grim scenario of an ever-emerging AMR burden (Willyard, 2017). Mutations to the regulator of the *mmpL5-mmpS5* genes (Rv0676c and Rv0677c) is responsible for BDQ resistance, as noted in previous studies. In this context, we examined compound **Il**’s efficiency in combination drug therapy with BDQ in a checkerboard assay, and fractional inhibitory concentrations were calculated. First, the MIC of BDQ against *M. abscessus* in MB7H9 and GAST media was determined to be between 0.43 μg/mL - 0.22 μg/mL and between 0.22 μg/mL - 0.05 μg/mL respectively. Following this, we investigated this new drug combination (BDQ and compound **Il**) against *M. abscessus* in both iron-rich and iron-deprived conditions. BDQ and compound **Il** combination showed additive profiles in both the medium against *M. abscessus* at their respective MICs. This newly identified drug combination by targeting essential (BDQ targets ATP synthase) and conditionally essential mechanism (mycobactin biosynthesis inhibition of compound **Il**) might pave the way to eradicate AMR challenges associated with superbugs in the coming days.

## Discussion

The emerging burden of AMR necessitates the discovery of EPIs as potential adjuvants in combination drug therapy to treat drug-resistant strains of mycobacteria. In the last decade, a significant number of approaches have been considered for the discovery of novel EPIs, but only a small number of EPI candidates are available for therapeutic intervention. In this study, we have identified compound **Il**, 2-(5-(4-fluorophenyl)-4, 5-dihydro-1H-pyrazol-3-yl)phenol as a potential whole-cell efflux pump inhibitor against *M. smegmatis* and *M. abscessus* strains of pathogen. Compound **Il** is the first rationally designed inhibitor that has been identified as an excellent mycobactin biosynthesis inhibitor against *M. abscessus*, which may pave the way for further inhibitors by disrupting the iron metabolism of a wide range of NTM. The HIT candidate is anticipated to bind to the MmpL4 and MmpL5’s mycobactin binding active site pocket since it partially resembles the siderophore mycobactin structure. Given the limited experimental structural determination for MmpL4/5 proteins, the putative binding pocket within MmpL4/5 was instead identified using an *in silico* approach. Further multi-omics studies will be required to validate MmpL4/5 as potential target for the novel compound **Il**. As previously highlighted, MmpL4/5 efflux pumps, other than siderophore transportation, are closely associated with bedaquiline resistance. To probe receptor changes in response to ligand binding, full atomistic MD simulations of the MmpL4/5 proteins embedded in a lipid bilayer and bound with compound **Il** were performed. The theoretical investigation into the ligand binding event predicted compound **Il** to be selective towards MmpL4, with the ligand maintaining significant hydrogen bonding and π-π stacking interactions with D272-Y845 and D844-Y273 residue pairs, effectively blocking the critical Asp-Tyr dyad interaction networks necessary for proton translocation. This novel drug combination of compound **Il** with bedaquiline highlighted an additive profile against the *M. abscessus* pathogen that may present a promising chemical framework for tackling emerging antibiotic resistance in NTM. Despite some promising discoveries in the recent decades, (Rodrigues et al., 2020; Laws et al., 2022) advancement in EPIs discoveries is encouraging to tackle the global burden of AMR using combination therapy. Thus, further explorations around the 3,5-diaryl-4,5-dihydro-1H-pyrazole chemical class could provide an encouraging solution to hasten the development of novel EPIs by reversing the efflux-pump-mediated antibiotic resistance in mycobacteria.

## Data availability statement

The original contributions presented in the study are included in the article/**Supplementary Material**. Further inquiries can be directed to the corresponding authors.

## Author contributions

MS: Formal analysis, Investigation, Methodology, Writing – original draft. AT: Formal analysis, Investigation, Methodology, Writing – original draft. CV: Formal analysis, Methodology, Software, Validation, Visualization, Writing – original draft, Writing – review & editing. CD: Formal analysis, Investigation, Methodology, Writing – original draft. OA: Conceptualization, Software, Supervision, Writing – review & editing. SB: Conceptualization, Funding acquisition, Project administration, Supervision, Writing – review & editing. VJ: Conceptualization, Funding acquisition, Project administration, Supervision, Writing – review & editing. 
