# Supplementary material for: Mycobactin analogue interacting with siderophore efflux-pump protein: insights from molecular dynamics simulations and whole-cell assays
Source: Front Antibiot. 2024 May 8;3:1362516. doi: 10.3389/frabi.2024.1362516 (PMC11731696; doi:10.3389/frabi.2024.1362516)
Supplement: Supplementary file 1 [file DataSheet_1.docx]

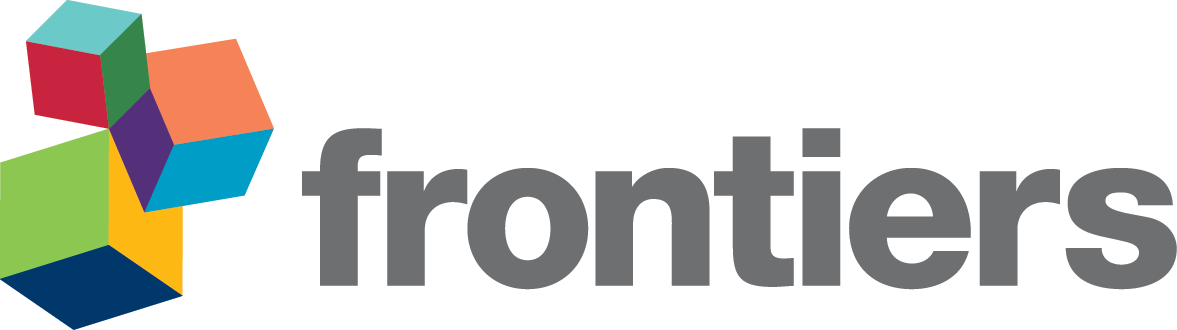


Supplementary Material

1. **SUPPLEMENTARY TABLES AND FIGURES**

# Tables

**Table S1.** Accelerated molecular dynamics boost potentials used in the MmpL4/5 simulations.

| System | | alphaD | alphaP | EthreshP | EthreshD |
| --- | --- | --- | --- | --- | --- |
|  | Orientation-1 | 1107 | 26650 | -309066 | 25850 |
| MmpL4 | Orientation-2 | 1098 | 26449 | -306809 | 25706 |
|  | Orientation-3 | 1126 | 27347 | -314774 | 26407 |
|  | Orientation-1 | 1133 | 27566 | -318390 | 26599 |
| MmpL5 | Orientation-2 | 1141 | 27789 | -322329 | 26758 |
|  | Orientation-3 | 1112 | 26896 | -314990 | 25982 |

# Figures





**Figure S1.** Comparing the different folded and unfolded regions by superimposing the MmpL4 protein structure predicted by AlphaFold (Grey) and Swiss-Model (Green)


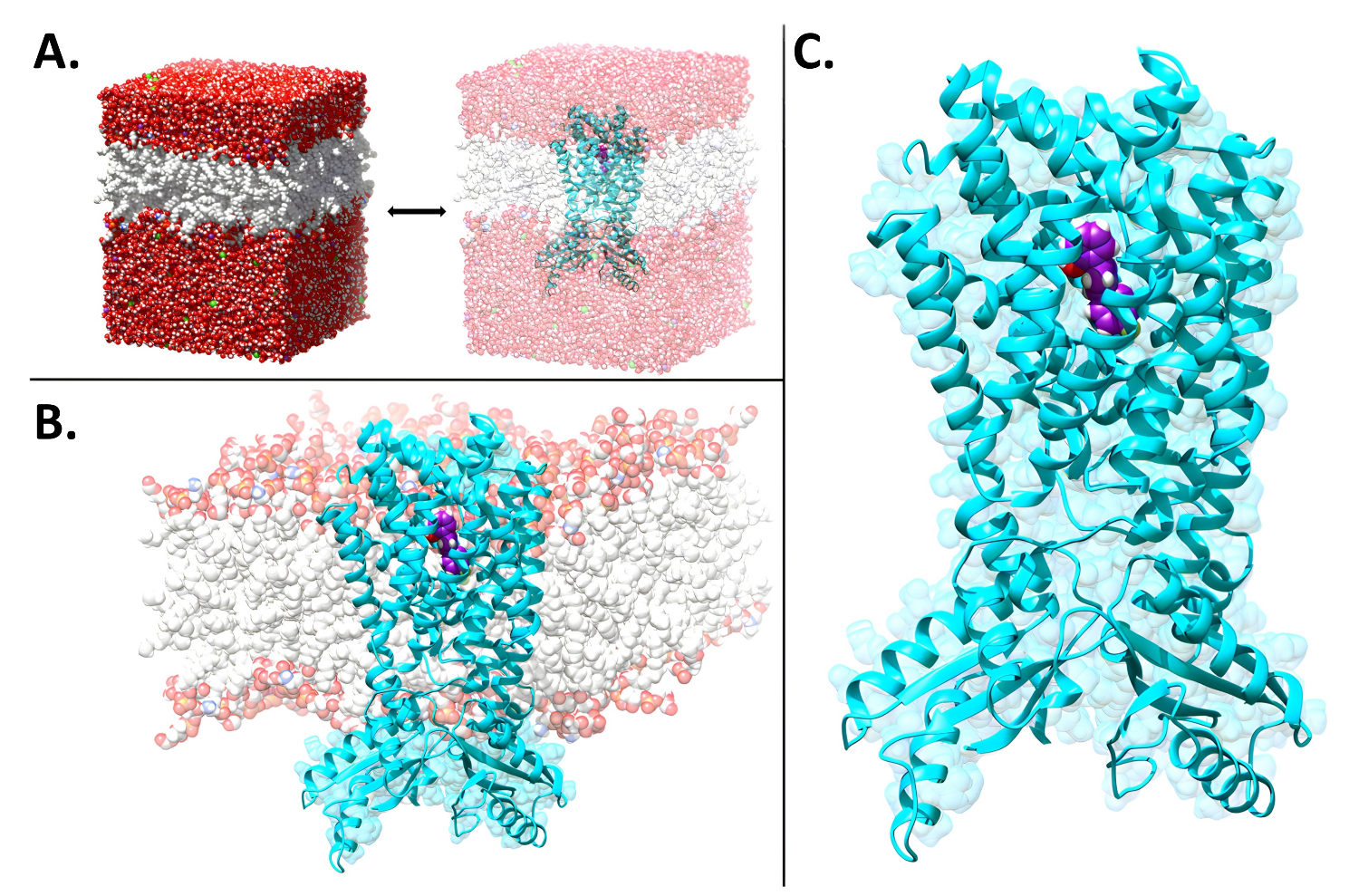


**Figure S2.** Graphical depiction of the lowest energy frame from the 100 ns aMD trajectory of the MmpL4-Il complex in orientation-3. (A) Visual representation of the MmpL4-Il complex, colored in *cyan* and *purple*, embedded in a lipid bilayer composed of 1-stearoyl-2-palmitoyl-sn-glycero-3-phopshoethanolamine (SPPE), 1,3-bis(1-oleoyl-2-palmitoyl-sn-glycero-3-phospho)-sn-glycerol (OPOPCL), 1-palmitoyl-2-oleoyl-sn-glycero-3-phosphoinositol (POPI), and 1-palmitoyl-2-myristoyl-sn-glycero-3-phosphoinositol (PMPI) lipids, colored *light* *gray*, in a 27:38:2:33 ratio respectively, and solvated with TIP3P water molecules and a 0.15 M KCl solution. Two representations of the fully solvated system are highlighted to showcase the location of the MmpL4-Il complex within the lipid membrane. (B) Visual representation of the MmpL4-Il complex, colored in *cyan* and *purple*, embedded in a SPPE:OPOPCL:POPI:PMPI lipid bilayer, colored *light gray*, with lipid heads colored *orange*, *red*, and *blue*. (C) Visual representation of the MmpL4-Il complex, colored *cyan* and *purple* respectively, sans the lipophilic microenvironment.


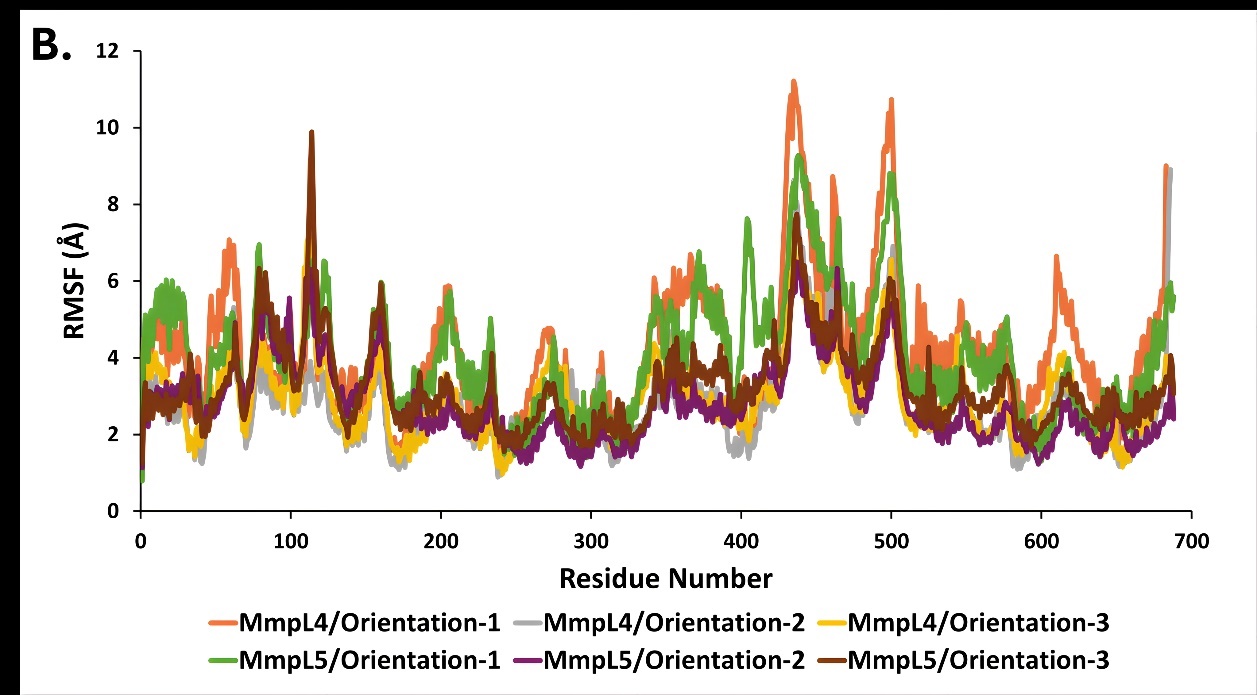

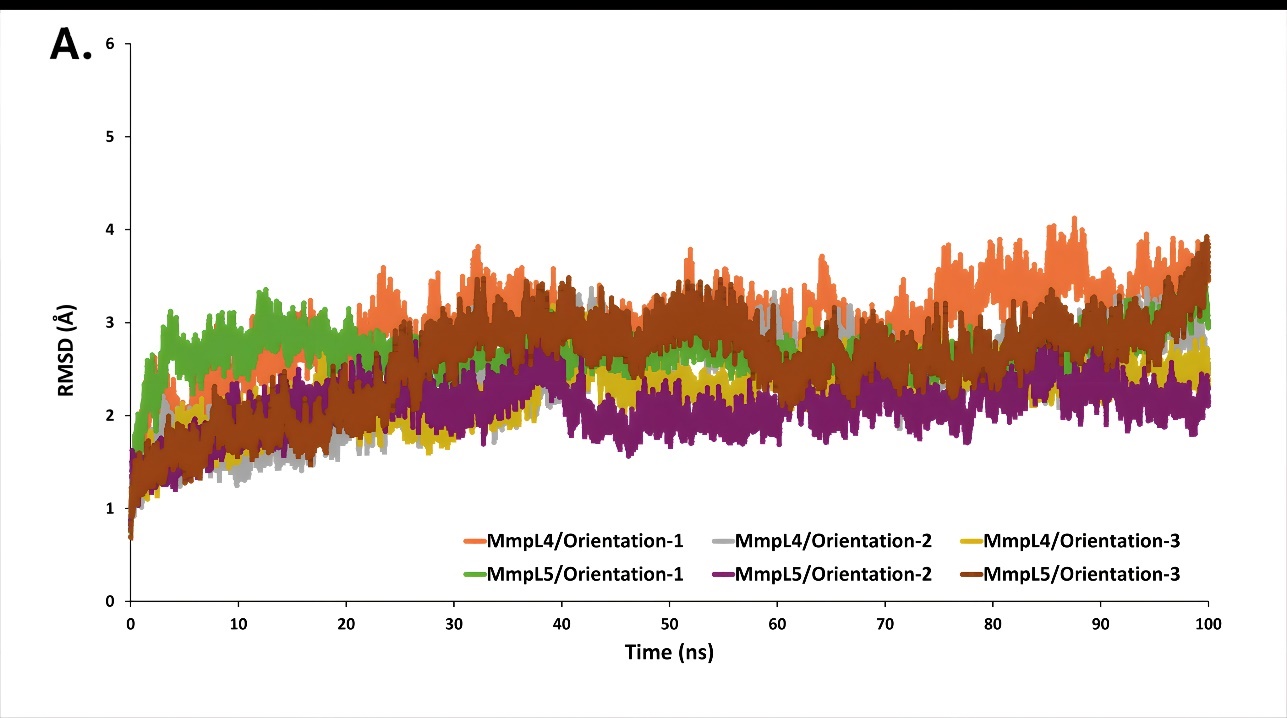


**Figure S3.** Root-mean-square deviations (RMSDs) relative to the first frame for the protein backbone atoms (N, C*_α_*, and C) of the MmpL4/5 receptors in the presence of compound Il in three different orientations bound at the putative active site. B) Root-mean-square fluctuations (RMSFs) by residue relative to the lowest energy structure computed over 100 ns of aMD for the MmpL4 and MmpL5 proteins backbone atoms (C, C*_α_*, and N) in the absence and presence of the Il inhibitor bound at the putative active site of MmpL4/5 in three different orientations.

**Figure S4.** Efflux-pump inhibition assay: Relative Fluorescence Unit (RFU) after 60 min: A. Data for compounds Ia-Im in M. smegmatis, and B. Data for compound Il in M. abscessus. VP- Verapamil (positive control), NC, NC1 and NC2 – DMSO (negative control). One-way anova followed by Tukey’s HSD using R-package. ns-not significant, **p < 0.01, ***p < 0.001, ****p < 0.0001, ns not significant.
